# SARS-CoV-2 variants evolved during the early stage of the pandemic and effects of mutations on adaptation in Wuhan populations

**DOI:** 10.7150/ijbs.47827

**Published:** 2021-01-01

**Authors:** Annoor Awadasseid, Yanling Wu, Yoshimasa Tanaka, Wen Zhang

**Affiliations:** 1 Lab of Chemical Biology and Molecular Drug Design, College of Pharmaceutical Science, Zhejiang University of Technology, Hangzhou, 310014, China.; 2 Lab of Molecular Immunology, Virus Inspection Department, Zhejiang Provincial Center for Disease Control and Prevention, Hangzhou, 310051, China.; 3 Department of Biochemistry & Food Sciences, University of Kordofan, El-Obeid, 51111, Sudan.; 4 Center for Medical Innovation, Nagasaki University, 1-7-1 Sakamoto, Nagasaki 852-8588, Japan.

**Keywords:** COVID-19, SARS-CoV-2, mutations, genomes, bioinformatics

## Abstract

The outbreak of the coronavirus disease 2019 (COVID-19) is caused by severe acute respiratory syndrome coronavirus 2 (SARS-CoV-2). The pandemic apparently started in December 2019 in Wuhan, China, and has since affected many countries worldwide, turning into a major global threat. Chinese researchers reported that SARS-CoV-2 could be classified into two major variants. They suggest that investigating the variations and characteristics of these variants might help assess risks and develop better treatment and prevention strategies. The two variants were named L-type and S-type, in which L-type was prevailed in an initial outbreak in Wuhan, Central China's Hubei Province, and S-type was phylogenetically older than L-type and less prevalent at an early stage, but with a later increase in frequency in Wuhan. There were 149 mutations in 103 sequenced SARS-CoV-2 genomes, 83 of which were nonsynonymous, leading to alteration in the amino acid sequence of proteins. Much effort is currently being devoted to elucidate whether or not these mutations affect viral transmissibility and virulence. In this review, we summarize the mutations in SARS-CoV-2 during the early phase of virus evolution and discuss the significance of the gene alterations in infections.

## Introduction

The coronavirus disease 2019 (COVID-19) pandemic started in December 2019 in Wuhan, Hubei Province, China. Since then, it has spread swiftly in China and many other countries worldwide, drawing major global attention 1. As of October 23, 2020, SARS-CoV-2 has infected more than 9,678,494 people worldwide, resulting in more than 1,143,357 deaths, with a mortality rate of 2.72% 2. The natural source of SARS-CoV-2 remains obscure. The initial cases were closely linked to a seafood market, suggesting the likelihood of a zoonotic infection 3. Although bats and pangolins are suspected to be hosts and intermediate hosts for wildlife, further investigation is required to support zoonotic infections and to trace the source of SARS-CoV-2 4-6. Coronavirus is an enclosed virus with a positive RNA genome, relating to the *Coronaviridae* family of the order *Nidovirales*, and is divided into four classes α, β, γ, and δ, in which SARS-CoV-2 belongs to the β genus 7. George Taiaroa et al. identified the first native SARS-CoV-2 RNA sequence, describing the coronaviral transcriptome and epitranscriptome and publicly disclosing those details 8. Coronaviruses have at least four essential proteins: spike (S), envelope (E), membrane (M), and nucleocapsid (N) proteins 9. S protein is glycosylated and supports host attachment and viral membrane fusion during viral infection. As a result, S protein somewhat determines the hosts' scope 7.

**Figure 1:**
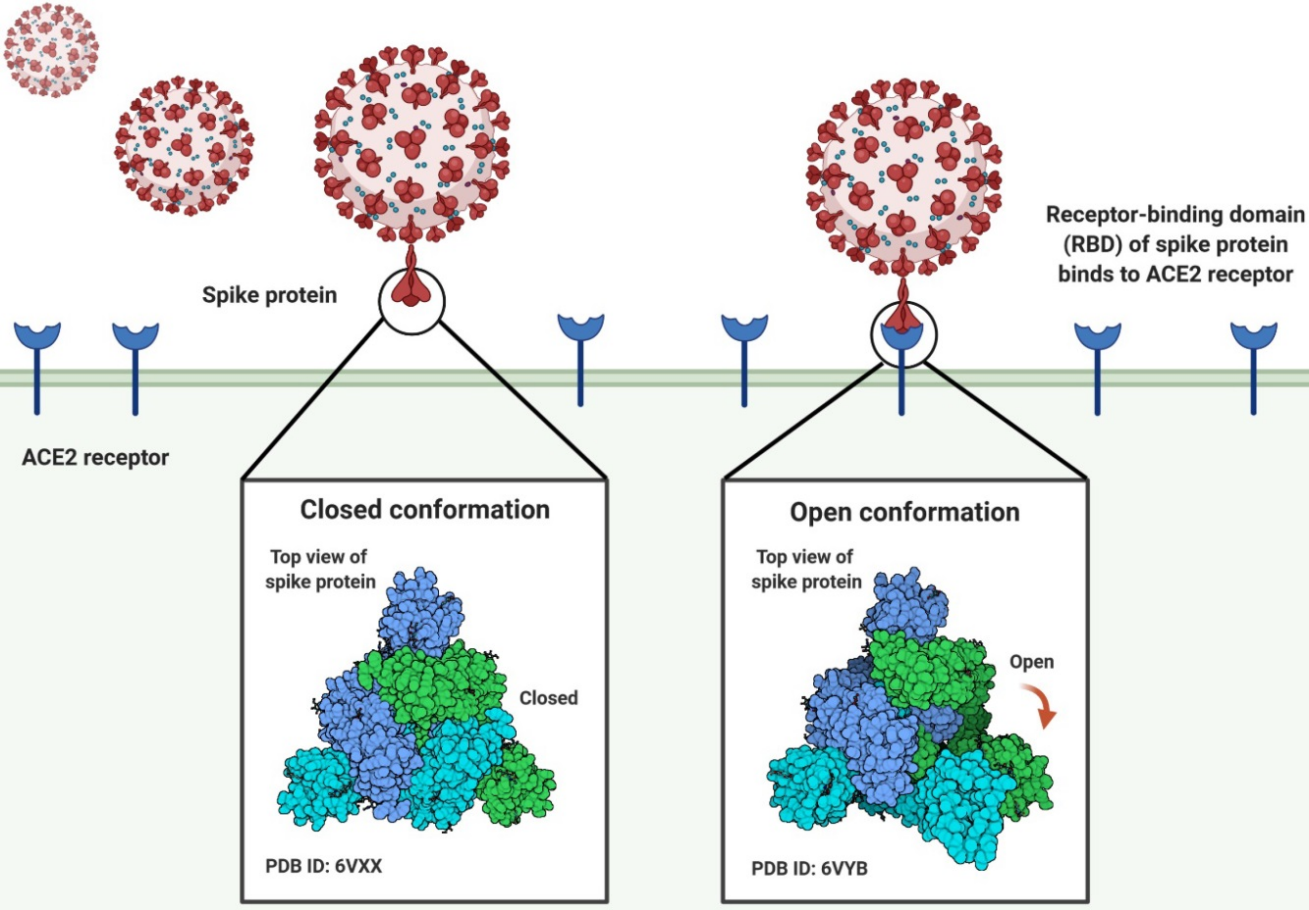
Three-dimensional conformation shifts of the spike protein of the SARS-CoV-2 virus as it binds to the human ACE2 receptor.

The angiotensin-converting enzyme 2 (ACE2) is the cellular receptor of SARS-CoV-2, which is identical to the receptor of SARS-CoV. When the virus infects cells, the S glycoprotein of SARS-CoV-2 recognizes and binds to ACE2. The S protein is composed of receptor binding subunit S1 and membrane fusion subunit S2. Previous studies have shown that S1 interacts with its receptors on the surface of host cells for viral attachment, while S2 fuses host and viral membranes, releasing the viral genome into host cells 10-13. The receptor-binding domain (RBD) of subunit S1 specifically interacts with ACE2, while the rest of the S protein does not. The RBD alone is enough to bind tightly to the peptidase domain of ACE2 (Fig. 1). Hence, RBD is thus a key determinant of virus-receptor interactions, virus-host range, tropism, and infectivity 10, 14, 15. Some studies have revealed that pangolins may implement a part of the spike gene for SARS-CoV-2. The key functional sites in the SAR-CoV-2 S protein are almost identical to the corresponding sites of viruses isolated from pangolins 5, 6, 16. Notwithstanding these latest findings, some major questions about the evolutionary patterns and driving forces behind the SARS-CoV-2 outbreak have not been addressed 17. Chinese experts studied the extent of molecular differences among SARS-CoV-2 and other coronavirus-related viruses and performed population genetic analysis on 103 sequenced genomes of SARS-CoV-2 (Table S1) 1. SARS-CoV-2 has mutated throughout the pandemic, resulting in changeable effects on COVID-19 and complicating attempts to control the outbreak 18. The SARS-CoV-2 mutation seems to be spreading globally and warrants special consideration.

## Mutation, recombination and transmission of coronaviruses

The mutation rates in human CoVs are moderate to high, when compared to those in other single-stranded RNA viruses, with the average substitution rates being about 10^-4^ substitutions per site each year 19. Recombination takes place when two or more related viruses invade the very same cell and leads to genetic differences in the offspring viruses, which may have an impact on the function of the host, virulence, host immune evasion, and antiviral resistance. Whereas 'antigenic shift' occurs in a segmented viral genome, like influenza virus genomes, certain 'recombination' mechanisms exist in unsegmented viruses 20. This is the case in viruses of the *Coronaviridae* family. Studies on another *Betacoronavirus*, Murine Hepatitis Virus (MHV), for instance, have shown that up to 25% of the progeny from co-infected cells had recombinant genomes. Haijema et al. reported that it took hours for the recombination to take place in feline infectious peritonitis virus (FIPV)-infected cat cells after injection with a gene fragment from MHV S protein, and that the resultant recombinant virus became contagious to mouse cells, but not to cat cells 21. The high recombination rates in CoVs are attributable to the particular mode of gene amplification in CoVs, termed discontinuous transcription based on the RNA-dependent RNA polymerase template-switching property 20.

The most common recombination breakpoints for SARS-CoV are inside the S protein-encoding gene enocoding the receptor-binding domain and the gene for an accessory protein 22. Previous studies on the relationship between the SARS-related CoV (SARSr-CoV) S protein and ACE2 demonstrate that several amino acid substitutions are required for the foreign S-protein to bind to the homologous receptor of the current host species 23-25. It was reported that SARS-CoV-2 also utilized ACE2 as its receptor for entry 26, 27. In addition, it should be noted that effective adaptation of CoVs to a new host needs not only such mutations affecting receptor binding, but also a complete set of positive gene mutations that improve the reproduction and transmission of viruses in the hew host 23.

Conventional human CoVs are transmitted mainly from humans to humans. Middle East respiratory syndrome (MERS) caused by MERS-CoV was, however, shown to occur occasionally with zoonotic transmission (from animals to humans) 28. Human CoVs are spread through direct interaction with secretions, fomites, and respiratory droplets 28. Human CoV disease is generally confined to the respiratory tract. However, Severe Acute Respiratory Syndrome coronavirus (SARS-CoV) is likely to propagate via both the fecal-oral path and the respiratory droplet/aerosol path, and stool has been found to be a valuable form of test for SARS-CoV diagnosis 29, 30. Such variability in the manner of transmission is also found in many domestic animals infected with wildlife-CoVs that may pass into humans because some of the wildlife-CoVs are enteric or pneumo enteric and released in feces (e.g., BCoV, PEDv, TGEV, FCoV, CCoV) 19, 31-33.

## SARS-CoV-2 genome mutations

**Figure 2:**
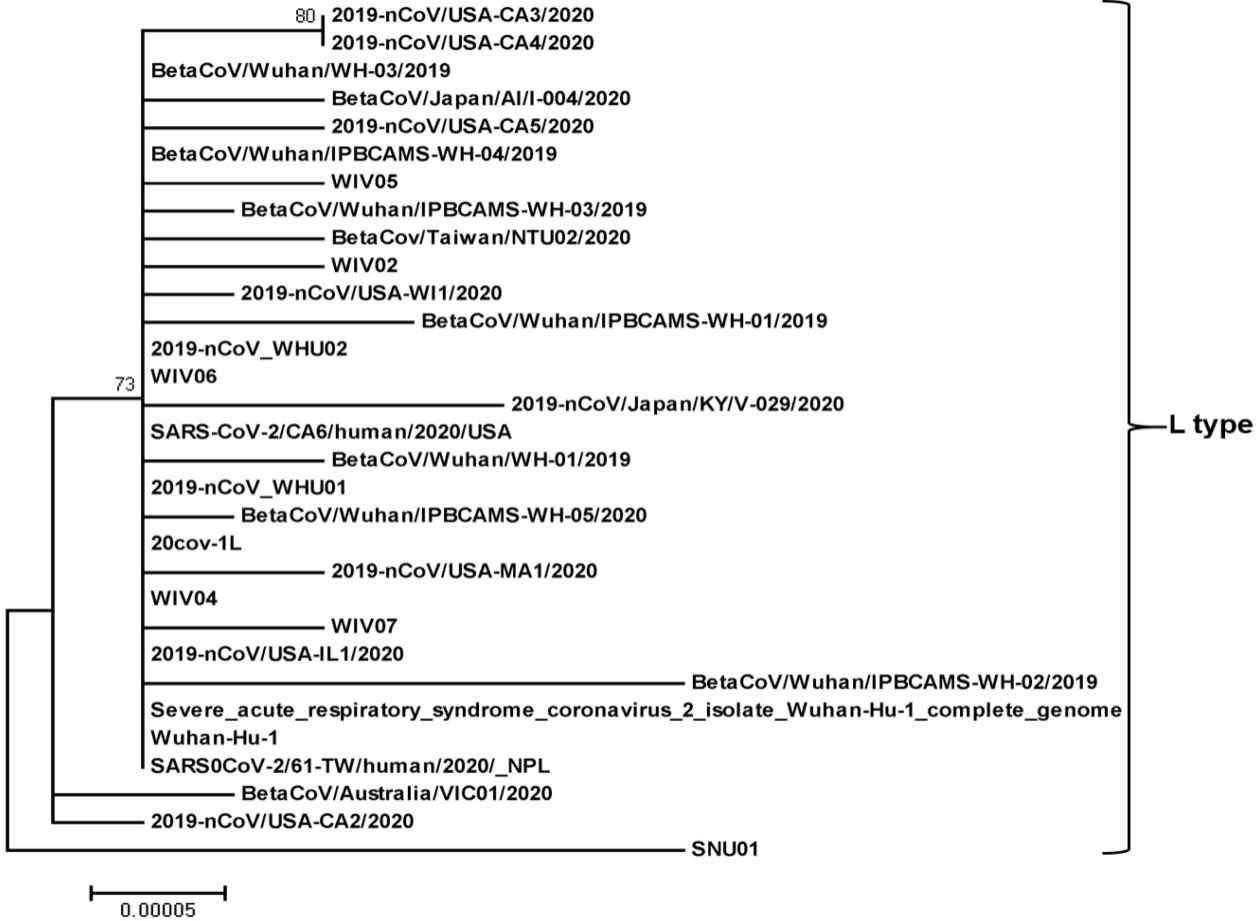
**Phylogenetic relationship of SARS-CoV-2-L-type.** The SARS-CoV-2-L-type full genome sequences were collected from the National Center for Biotechnology Information search engine (http:/www.ncbi.nim.nih.gov/). The phylogenetic tree was built with 1000 bootstrapped value help and a Poisson correction utilizing the MEGA 5.0 software package and neighbor-joining program (http:/www.megasoftware.net). The bootstrap values are provided at nodes higher than 50%. The scale bar displays the range of phylogenetic variations calculated from the number of changes. The genome sequence accession numbers at NCBI GenBank are MT027062 (2019-nCoV/USA-CA3/2020), MT027063 (2019-nCoV/USA-CA4/2020), LR757996 (BetaCoV/Wuhan/WH-03/2019), LC521925 (BetaCoV/Japan/AI/I-004/2020), MT027064 (2019-nCoV/USA-CA5/2020), MT019532 (BetaCoV/Wuhan/IPBCAMS-WH-04/2019), MN996529 (WIV05), MT019531 (BetaCoV/Wuhan/IPBCAMS-WH-03/2019), MT066176 (BetaCov/Taiwan/NTU02/2020), MN996527 (WIV02), MT039887 (2019-nCoV/USA-WI1/2020), MT019529 (BetaCoV/Wuhan/IPBCAMS-WH-01/2019), MN988669 (2019-nCoV_WHU02), MN996530 (WIV06), LC522972 (2019-nCoV/Japan/KY/V-029/2020), MT044258 (SARS-CoV-2/CA6/human/2020/USA), LR757998 (BetaCoV/Wuhan/WH-01/2019), MN988668 (2019-nCoV_WHU01), MT019533 (BetaCoV/Wuhan/IPBCAMS-WH-05/2020), MT039873 (20cov-1L), MT039888 (2019-nCoV/USA-MA1/2020), MN996528 (WIV04), MN996531 (WIV07), MN988713 (2019-nCoV/USA-IL1/2020), MT019530 (BetaCoV/Wuhan/ IPBCAMS-WH-02/2019), NC_045512 (Severe_acute_respiratory_syndrome_ coronavirus_2_ isolate_Wuhan-Hu-1_complete_genome), MN908947 (Wuhan-Hu-1), MT072688 (SARS0CoV-2/61-TW/human/2020/_NPL), MT007544 (BetaCoV/Australia/VIC01/2020), MN994468 (2019-nCoV/USA-CA2/2020), and MT039890 (SNU01).

**Figure 3:**
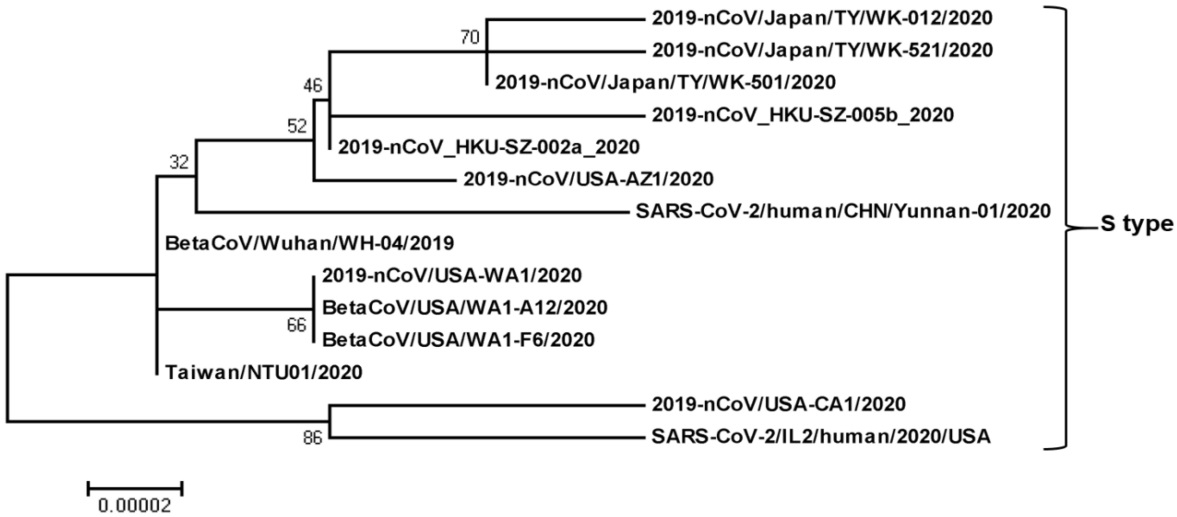
** Phylogenetic relationship among SARS-CoV-2-S-type genomes.** The SARS-CoV-2-S-type full genome sequences were collected from the National Center for Biotechnology Information search engine (http:/www.ncbi.nim.nih.gov/). The phylogenetic tree was built with 1000 bootstrapped value help and a Poisson correction utilizing the MEGA 5.0 software package and neighbor-joining program (http:/www.megasoftware.net). The bootstrap values are provided at nodes higher than 50%. The scale bar displays the range of phylogenetic variations calculated from the number of changes. The genome sequence accession numbers at NCBI GenBank are LC522973 (2019-nCoV/Japan/TY/WK-012/2020), LC522975 (2019-nCoV/Japan/TY/WK-521/2020), LC522974 (2019-nCoV/Japan/TY/WK-501/2020), MN975262 (2019-nCoV_HKU-SZ-005b_2020), MN938384 (2019-nCoV_HKU-SZ-002a_2020), MN997409 (2019-nCoV/USA-AZ1/2020), MT049951 (SARS-CoV-2/human/CHN/Yunnan-01/2020), LR757995 (BetaCoV/Wuhan/WH-04/2019), MN985325 (2019-nCoV/USA-WA1/2020), MT020880 (BetaCoV/USA/WA1-A12/2020), MT020881 (BetaCoV/USA/WA1-F6/2020), MT066175 (Taiwan/NTU01/2020), MN994467 (2019-nCoV/USA-CA1/2020), and MT044257 (SARS-CoV-2/IL2/human/2020/USA).

Recently, a total of 149 mutations have been found in 103 sequenced strains evolved in the early stage of the pandemic (Table S1, Fig. 2, 3). The ancestral states of 43 synonymous, 83 non-synonymous, and two terminating gain mutations were explicitly indicated 1. The greatest of the derived mutations were 67.4% of synonymous mutations and 84.3% of non-synonymous mutations, showing new origin or population growth 34, 35. Non-synonymous mutations in alleles obtained from at least two SARS-CoV-2 strains affected six proteins: S (H49Y, and V367F), N (S194L, S202N, and P344S), ORF3a (G251V), ORF7a (P34S), ORF8 (V62L, and S84L), and orf1ab (A117T, I1607V, L3606F, and I6075T) 1. Through population genetic analysis of 103 genomes of SARS-CoV-2, SARS-CoV-2 developed into two main types (L and S) in the early stage of the pandemic, which are well represented by only two almost complete single nucleotide polymorphisms (SNPs) linkage between SARS-CoV-2 strains 1. The genomic average (synonymous replacements per synonymous site) dS value within SARS-CoV-2 and Guangdong (GD) Pangolin-CoV was 0.475, which is comparable to that between humans and mice (0.5), and even higher (0.722) between SARS-CoV-2 and Guangxi (GX) Pangolin-CoV (Table 1) 36. The extent of these measures implies that variations in neutral evolutionary sites rather than changes in all nucleotide sequences can be used for the determination of the source and natural intermediate hosts of SARS-CoV-2.

Peng Zhou et al. observed that SARS-CoV-2 S protein associates with human ACE2, which facilitates the entry of SARS-CoV-2, which means that human ACE2 is the SARS-CoV-2 receptor 4. ACE2 comprises at least five essential amino acids for binding the SARSr-CoV S protein 15. Junwen Luan et al. examined the related amino acids of various mammals based on these five amino acids to decide which mammalian ACE2 may associate with the human SARSr-CoV S protein 37. Through studying the protein sequence of mammalian ACE2, they noticed that the ACE2 of *Camelus dromedarius*, *Procyon lotor*, *Rhinolophus ferrumequinum*, *Rattus norvegicus*, *Mus musculus*, *Ornithorhynchus anatinus*, *Loxodonta africana*, *Erinaceus europaeus*, *Nyctereutes procyonoides*, *Suricata suricatta*, *Dipodomys ordii*, and *Cavia porcellus* cannot interact with S protein 37. Such species may be removed from the possible SARS-CoV-2 host list. They observed that certain wild mammals might bind S protein to ACE2, indicating that we would investigate whether those species could be intermediate hosts for SARS-CoV-2 37. The receptor-binding motif (RBM) domain in the S protein of pangolin coronavirus has been documented to be identical to that of the SARS-CoV-2 S protein 38, 39, which could be implicated in the recombination of SARS CoV-2. They noticed that N82 of pangolin ACE2 displayed more significant interaction with RBD than human ACE2, suggesting that pangolin ACE2 might have a stronger association with SARS-CoV-2 37. This observation also confirms the assumption that pangolin plays a part in the development of SARS CoV-2.

### Two SARS-CoV-2 variants evolved in the early stage of the pandemic

Chinese researchers initially determined SARS-CoV-2 L and S variants in terms of two closely related SNPs. When they reconstructed haplotype networks utilizing all SNPs in the SARS-CoV-2 genome, the separation of L and S types was observed and the two associated SNPs at sites 8,782 and 28,144 fully determined the L and S types of SARS-CoV-2 1. To define whether type L or type S is ancestral, they compared genomes among SARS-CoV-2 and closely related viruses. Surprisingly, the S-type nucleotides at sites 8,782 and 28,144 were similar to the right homologous locations in the several nearly related viruses. Notably, both sites are highly conserved among other viruses. Thus, although type L variant (about 70%) was more common than type S variant (about 30%) in the SARS-CoV-2 they tested, type S was the old version of SARS-CoV-2 1. The mutation load analysis showed that L type accumulated more derivative mutations than S type. Although L type is a new evolution from the old S type, it spreads or replicates readily in Wuhan, increasing more mutations than the S type. L type thus seems to be more adopted in Wuhan populations than S type. It is, however, uncertain whether or not there is a difference in transmissibility and virulence between the two variants.

To verify whether there were variations in the temporary and spatial arrangement of the two types of SARS-CoV-2, they stratified the virus according to the isolated location and date. With the 27 viruses isolated from Wuhan, L type accounted for 96.3%, and S type accounted for 3.7%. Nevertheless, of the other 73 viruses isolated outside Wuhan, 61.6% comprised of L type, and 38.4% consisted of S type. This illustration shows that L type is more common in Wuhan than in other cities 1. As of January 2020, the Chinese government has taken quick and extensive preventive and control plans. These personal mediations can lead to severe selection pressures for L type, which seemed to be more adopted in Wuhan populations. On the other hand, due to personal mediation, the particular pressure of S type might be weak, increasing its comparative abundance in the SARS-CoV-2. The two SARS-CoV-2 variants, therefore, might be subject to various selection pressures depending on their epidemiological characteristics 1. Notably, the above analysis was based on very scattered SARS-CoV-2 genomes obtained from various places and periods. Larger genomic data is thus needed to examine the hypothesis further. Yet, it is not clear whether L type developed from S type in humans or intermediate hosts. It is imperative to implement further studies for the elucidation of the relationship between the mutations and transmissibility and virulence.

## SARS-CoV-2 mutation rate during the early stage of the pandemic

The genome of SARS-CoV-2 has been deemed genetically more stable than that of SARS-CoV or MERS-CoV until now 40. However, depending on the genome sequence evidence presently available, the SARS-CoV-2 mutation risk is significantly similar to SARS, which triggered the epidemic in 2002-2003 1, 41. Previous studies have suggested the genomes of SARS-CoV-2 are very homogeneous. Molecular geneticists who closely track the virus's evolution have proposed that the SARS-CoV-2 mutation rate would remain low 41, 42. Although it is usually reasonable to assume that SARS-CoV-2 continues to mutate at a low rate, all existing analyses focus solely on early-stage data obtained from this pandemic 41. The development and mutation dynamics of SARS-CoV-2 also need to be carefully studied, as the virus continues to propagate quickly across the world, and more genomic evidence is accumulating 41. Yong Jia et al. discovered that in the phylogenetic tree's center with the shortest branch, the earliest few recorded SARS-CoV-2 accessions obtained from Wuhan China were identified. Interestingly, various U.S. viral genomes have been identified, almost similar to the putative initial variants of Wuhan viral 41.

Roujian Lu et al., first found that the S-protein RBD in SARS-CoV-2 is related to human SARS-CoV while the other part of its genome is much more analogous to SARS-CoV bat 43. Tommy Tsan-Yuk Lam et al. later described a CoV RaTG13 bat and many SARS-CoV pangolins, which are significantly similar to SARS-CoV-2 than human SARS-CoV in either full-S or RBD protein 44. Depending on SARS-CoV-2's close association to SARS, SARS-CoV-2 vaccines and medicines' ongoing production has also concentrated on the S protein and its human binding receptor ACE2 45, 46. Observation by Yong Jia et al. raised the alarm that SARS-CoV-2 mutation with a varying epitope phenotype may occur at any moment, which suggests that the existing production of the vaccine against SARS-CoV-2 is at high risk of being ineffective. Since the receptor identification process between SARS-CoV-2 and SARS-CoV, which has been shown to share the specific human cell receptor ACE2, it seems strongly conserved 41. One recommendation for the next phase in drug discovery is likely to concentrate on discovering possible human ACE2 blocker receptors, as indicated in a recent statement 45. This strategy would overcome the aforementioned threat to the development of vaccines.

Hangping Yao et al., three results stood out in their study: first, in the 11 viral isolates, a vast array of mutations was reported, including two sets of mutations forming two main clusters of viruses presently able to infect the global population. Moreover, given the comparatively early sampling dates, 19 of the 31 mutations found are new, suggesting that the true variety of viral strains is still mostly undervalued; second, significantly the mutations T22303G and A22301C result in the same S247R mutation in the S-protein, and mapping the current structure showed that this residue is situated in a stable loop area inside the N-terminal domain of the S-protein subunit S1. However, the precise location of S247 could not be established 47. Although the N-terminal domain is not explicitly related to ACE2 48, Hangping Yao et al. states that this domain is situated right next to the C-terminal domain, which connects to ACE2. Surprisingly, the T22303G mutation was found in 5 viral isolates, although in specific amounts, suggesting that this particular mutation was still present throughout the early days of the pandemic, and possibly in a small number of Wuhan citizens, given the fact that it is still mostly absent from the current GISAID database 47. It may be attributable to the mutation's founding influence, in which case during the early days the T22303G mutation was not transmitted from China 47; third, the tri-nucleotide mutation in ZJU-11 is unanticipated; they recognize that in their viral load and Cytopathic effects (CPE) assay this particular viral isolate is very active, and their patient stayed positive for an impressive 45-days period and was just recently released from the hospital 47. This will be particularly important to investigate the practical effect of this tri-nucleotide mutation. They notice that a further tri-nucleotide mutation (G28881A, G2882A, and G28883C) has been found in the existing collection, which also contributes to two protein-level missense mutations. It contributes to a cluster of over 300 viral strains, and it would be worth studying their mutational effect on viral pathogenicity 47. Eventually, in comparison to the recent study that a viable viral isolate could not be collected from faecal samples, three of their isolated viral samples were obtained from faeces samples 47, suggesting that the SARS-CoV-2 would reproduce in faecal samples 49.

Yvonne CF Su et al. identified the first significant biological occurrence of the SARS-CoV-2 virus since its introduction into the human community 40. While the biological effects of this deletion are unclear, this could affect the virus phenotype owing to the modification of the N gene transcription 40. Previous research has suggested that SARS-CoV's ORF8 plays a specific role in replicative fitness viruses and can be correlated with attenuation during the initial stages of human-to-human transmission 50. Given the occurrence of several deletions in SARSr-CoV's ORF8, it is possible that with the continued transmission of SARS-CoV-2 in humans, we may see more forms of deletion evolving 40. Potential work will concentrate on the phenotypic impact of **Δ**382 viruses on global disease propagation mechanisms and the immediate application of this genomic marker to molecular epidemiological science 40.

## ACE2 conservation and its ability to be used by SARS-CoV-2 as a receptor

**Figure 4:**
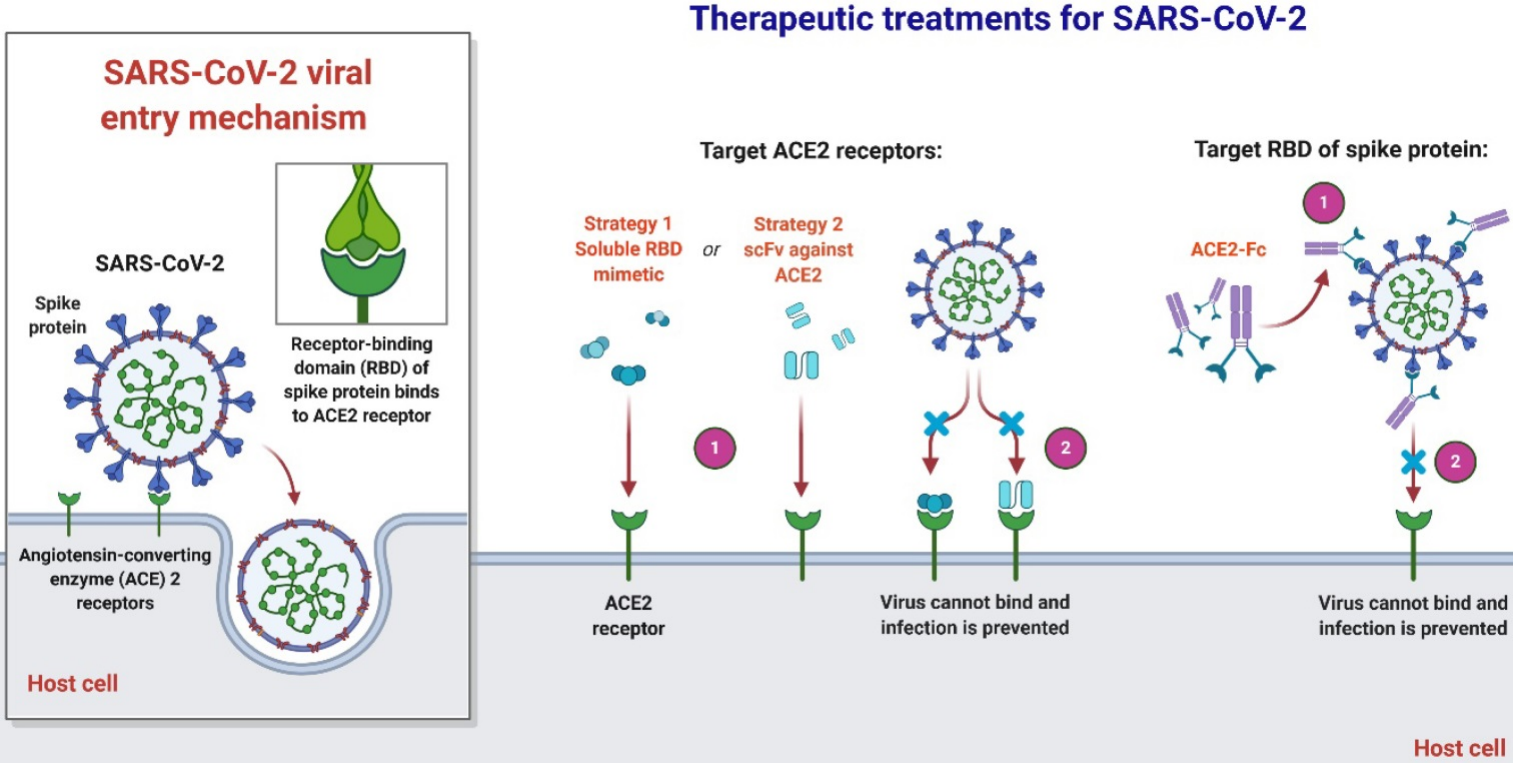
An overview of therapeutic strategies to treat SARS-CoV-2 infection based on virus-cell interaction. Host-targeted strategies include RBD mimetics and antibody fragments, such as scFv. Virally-targeted strategies include antibodies or antibody fragments, such as Fc. In both cases, the ACE2-RBD interaction is inhibited, preventing infection.

The phylogenetic study of coronaviruses has shown that SARS-CoV-2's immediate ancestor quite probably evolved from a bat organism 4. Nevertheless, it is still not determined if SARS-CoV-2 or a progenitor of this virus has been transmitted directly to humans or via an intermediate host. Joana Damas et al. conducted comprehensive comparative genomics, evolutionary and structural study of ACE2, which acts as the SARS-CoV-2 receptor in humans, to classify potential intermediate host species and species at risk SARS-CoV-2 infection (Fig. 4) 51. Previous studies have drawn on the increasing global database of annotated genomes of vertebrates, particularly new genomes provided by the Bat1K Collaboration, Zoonomia, and Vertebrate Genomes Project, associated with Genomes 10K-affiliated, as well as other sources 52, 53. A phylogenetic study of ACE2 orthologs from 410 vertebrates was performed. Their ability to bind SARS-CoV-2 S was estimated using a calculation dependent on amino acid residues at 25 binding residues of consensus human ACE2 54, 55. For the prediction of cross-species transmission of viruses, like SARS-CoV, similarity-based methods are commonly used 56-58. Joana Damas et al. validated these hypotheses with a detailed structural study of the SARS-CoV-2 S complexed ACE2 binding site. They also examined the assumption that in mammalian lineages with various predispositions to coronaviruses, the ACE2 receptor is subject to selective restrictions.

Joana Damas et al. expect that organisms with a very high SARS-CoV-2 S binding to ACE2 tendency would be extremely likely to become infected with the virus and could be possible intermediate hosts for virus transmission. As well as suggesting that several species with a medium score have an absolute chance of infection, species with a very low or low score are less susceptible to infection with SARS-CoV-2 through the ACE2 receptor 51. Notably, their assumptions are dependent exclusively on in-silico studies and should be checked by relevant analytical outcomes. As more comprehensive data are produced demonstrating the effect of ACE2 mutations on its ligand binding for SARS-CoV-2 S, which might require knowledge-based measurement of residues in the scoring algorithm, the model's estimation reliability could be enhanced. Until the developed simulation precision can be checked with subsequent experimental evidence, they advise precaution not to over-interpret the current study's predictions. In terms of species, threatened or otherwise, this is particularly critical in human treatment, although high or medium-ranked species may be prone to infection based on their ACE2 residues' characteristics 51. Clinical results throughout species vary much based on other processes, such as immune responses, which may influence the viral replication and propagate to appropriate cells, tissues, and organs. In addition, the probability that infection happens in any species through another cellular receptor, as seen for many other beta-coronaviruses, or interactions of lower affinity with ACE2 as suggested for SARS-CoV, could not be excluded 26, 56, 59. Nevertheless, their hypotheses provide a valuable baseline for selecting suitable animal models for the study of COVID-19 and detecting species that could be at risk of SARS-CoV-2 transmission from human to animal or from animal to animal.

The function of ACE2 in SARS-CoV-2 binding and cellular infection and its association with laboratory and natural diseases in various species have been investigated in many previous studies 26, 37, 60-63. Joana Damas et al. design differs significantly from those in many aspects: (I) a greater number of primates, carnivores, rodents, cetartiodactyls, and other mammalian orders were examined, as well as comprehensive phylogenetic analysis of fishes, birds, amphibians, and reptiles; (II) the complete range of S-binding residues in the ACE2 binding site was evaluated based on a consent range out in two independent studies 54, 55; (III) in assessing the ACE2 binding potential for SARS CoV-2 S, they used various methodologies; and (IV) their research evaluated the whole ACE2 protein for selection and rapid development. Although their findings are compatible with the findings and conclusions of Melin Amanda D et al. 62 on the hypothesized vulnerability of primates to SARS-CoV-2, especially Old-World primates, assumptions were provided for a greater number of primates (*n* = 39 vs. *n* = 27), bats (*n* = 37 vs. *n* = 7), various mammals (*n* = 176 vs. *n* = 5) as well as other vertebrates (*n* = 158 vs. *n* = 0). There were several similarities when comparing ACE2 from species in their analysis with other research findings, such as the low risk for rodents. However, some assumptions differ, including the comparatively high risk expected by others for pangolin and horse SARS-CoV-2 S binding 63, civet 15, Chinese rufous horseshoe bat 15, and turtles 64. Their findings are broadly similar to research that examined the binding affinity of soluble ACE2 with saturated mutations for SARS-CoV-2 S RBD, especially in the binding hot-spot area of ACE2 residues 353 to 357 65. Notably, their findings significantly increased the list of potential intermediate hosts relative to other reports. They established several new endangered species which might be at risk of SARS-CoV-2 infection through their ACE2 receptors.

The serious dispute surrounds claims that pangolins may act as a SARS-CoV-2 intermediate host, with certain findings suggesting that SARS-CoV-2 originated as a recombinant among bat and pangolin betacoronaviruses 66, 67, whereas another research refuted that assertion 68. ACE2 for Chinese pangolin, Sunda pangolin, and white-bellied pangolin seemed to have a slight or feeble binding rate for SARS-CoV-2 S. Utilizing molecular binding models, binding of pangolin ACE2 to SARS-CoV-2 S was anticipated 67. Nevertheless, neither laboratory nor *in vitro* SARS-CoV-2 infection was documented for pangolins. To determine whether SARS-CoV-2 S binds to pangolin ACE2, more investigations are required. Melin Amanda D et al. have shown that all primates, such as chimpanzees, bonobos, gorillas, orangutans, and all African and Asian primates (catarrhines) have the identical set of 12 primary residues of amino acids as human ACE2. In the Americas, monkeys and some tarsiers, lemurs, and lorisoids differ in important interaction residues, and protein modeling suggests that these variations would substantially decrease the binding affinity of ACE2 to the virus, thus moderating their vulnerability to infection 62. It is expected that other lemurs are similar to catarrhines in their vulnerability. Melin Amanda D et al. indicated that, and perhaps several lemurs, monkeys, and African and Asian monkeys are all prone to be particularly vulnerable to SARS-CoV-2, posing a crucial threat to their survival. In order to restrict the exposure of Great Apes to humans, immediate steps have been taken, and comparable attempts will be required for several other primate species.

## Conclusion

Researchers recently suggested that 103 SARS-CoV-2 strains evolved during the early phase of outbreak in Wuhan might be classified into two main types called L and S, with L variants being more predominant and comprising 70% of the strains tested. Whereas S variants are the ancestral strains, L variants seem to be more adapted in Wuhan populations than their ancestors 1. Furthermore, according to later studies, the L-type is slightly more widespread in Wuhan than elsewhere. After January 2020, however, the proportion of L variants was declined relative to that of S variants and the outbreak of SARS-CoV-2 has been slowed down in Wuhan 1. It was hypothesized that this might be attributed to the swift and extensive preventive steps being taken by Chinese central and local governments that created extreme selection pressure against L variants. Nevertheless, they added, the hypothesis needs more careful and extensive verification 1. Scientists have noticed that many patients were infected with either L or S variants of SARS-CoV-2, but there could be further mutations as the pandemic proceeds. For instance, a 63-year-old female patient in Chicago was infected with both L and S types of SARS-CoV-2 strains after she traveled in Wuhan and returned to the United States on Jan 13, 2020. Furthermore, a patient in Australia was found to carry at least two strains of SARS-CoV-2 when he returned from China. Such cases represent the emerging complexity of SARS-CoV-2 infections 1. It would be of great interest to continue research exploring how the different SARS-CoV-2 viral alleles interact among each other.

It is much too early to conclude that the virus has mutated into something more dangerous or more benevolent because all we understand is that the mutations can appear on a portion of the genome, which will do nothing. The longer we study the virus, the more confidence we unraveled. An important question that we should address next is whether the strains found in non-symptomatic carriers are S or L variants, or totally different mutants. Recent reports indicated that L variants underwent further mutation and were divided into two explicitly different subtypes outside of China 69. In this report, the original S variants correspond to A types and L variants to B and C types, in which C variants were evolved from B variants. Taken together, SARS-CoV-2 is being mutated even now and we have to continue to monitor the emergence of more transmissive and virulent strains of SARS-CoV-2.

## Supplementary Material

Supplementary table S1.

## Figures and Tables

**Table 1:** SARS-CoV-2 and other related viruses

Accession ID	Virus name	Simplified names	Databases
NC_045512	BetaCoV/Wuhan-Hu-1/2019	SARS-CoV-2	Genbank
MN996532	BetaCoV/bat/Yunnan/RaTG13/2013	Bat RaTG13	Genbank
MG772934	Unknown	Bat SARSr-CoV ZXC21	Genbank
MG772933	Unknown	Bat SARSr-CoV ZC45	Genbank
NC_004718	Unknown	SARS-CoV	Genbank
NC_014470	Unknown	Bat SARSr-CoV BM48-31	Genbank
EPI_ISL_410721	BetaCoV/pangolin/Guandong/1/2019	GD Pangolin-CoV	GISAID
EPI_ISL_410538	BetaCoV/pangolin/Guangxi/P4L/2017	GX Pangolin-CoV_P4L	GISAID
EPI_ISL_410539	BetaCoV/pangolin/Guangxi/P1E/2017	GX Pangolin-CoV_P1E	GISAID
EPI_ISL_410540	BetaCoV/pangolin/Guangxi/P5L/2017	GX Pangolin-CoV_P5L	GISAID
EPI_ISL_410541	BetaCoV/pangolin/Guangxi/P5E/2017	GX Pangolin-CoV_P5E	GISAID
EPI_ISL_410542	BetaCoV/pangolin/Guangxi/P2V/2017	GX Pangolin-CoV_P2V	GISAID
EPI_ISL_410543	BetaCoV/pangolin/Guangxi/P3B/2017	GX Pangolin-CoV_P3B	GISAID
