# Supplementary material for: SARS-CoV-2 variants evolved during the early stage of the pandemic and effects of mutations on adaptation in Wuhan populations
Source: Int J Biol Sci. 2021 Jan 1;17(1):97–106. doi: 10.7150/ijbs.47827 (PMC7757051; doi:10.7150/ijbs.47827)
Supplement: Supplementary file 1 — Supplementary table S1. [file ijbsv17p0097s1.pdf]

Supplementary Materials

SARS-CoV-2 variants evolved during the early stage of the pandemic and effects of mutations on adaptation in Wuhan populations

Annoor Awadasseid[1, 3], Yanling Wu[2✉], Yoshimasa Tanaka[4], Wen Zhang[1✉]

1 Lab of Chemical Biology and Molecular Drug Design, College of Pharmaceutical Science, Zhejiang University of Technology, Hangzhou, 310014, China.

2 Lab of Molecular Immunology, Virus Inspection Department, Zhejiang Provincial Center for Disease Control and Prevention, Hangzhou, 310051, China.

3 Department of Biochemistry & Food Sciences, University of Kordofan, El-Obeid, 51111, Sudan.

4 Center for Medical Innovation, Nagasaki University, 1-7-1 Sakamoto, Nagasaki 852-8588, Japan.

✉ Corresponding authors: Yanling Wu, Lab of Molecular Immunology, Virus Inspection Department of Zhejiang Provincial Center for Disease Control and Prevention, 630 Xincheng Road, Hangzhou, 310051, PR China; Tel: +86-571-87115282; Fax: +86-571-87115282; e-mail: ylwu@cdc.zj.cn. Wen Zhang, Lab of Chemical Biology and Molecular Drug Design, College of Pharmaceutical Science, Zhejiang University of Technology, 18 Chaowang Road, Hangzhou, 310014, PR China; Tel: +86-571-88871507; Fax: +86-571-88871507; e-mail: wzhang63@zjut.edu.cn.

Table S1. Genetic study of the population on 103 sequenced SARS-CoV-2 genomes.

Accession ID	Virus name	Subtype	Databases
EPI_ISL_406844	BetaCoV/Australia/VIC01/2020	L	GISAID
EPI_ISL_408481	BetaCoV/Chongqing/IVDC-CQ-001/2020	L	GISAID
EPI_ISL_408479	BetaCoV/Chongqing/ZX01/2020	L	GISAID
EPI_ISL_406597	BetaCoV/France/IDF0373/2020	L	GISAID
EPI_ISL_406596	BetaCoV/France/IDF0372/2020	L	GISAID
EPI_ISL_408431	BetaCov/France/IDF0626/2020	L	GISAID
EPI_ISL_408430	BetaCoV/France/IDF0515/2020	L	GISAID
EPI_ISL_406534	BetaCoV/Foshan/20SF207/2020	L	GISAID
EPI_ISL_406535	BetaCoV/Foshan/20SF210/2020	L	GISAID
EPI_ISL_406536	BetaCoV/Foshan/20SF211/2020	L	GISAID
EPI_ISL_403934	BetaCoV/Guangdong/20SF014/2020	L	GISAID
EPI_ISL_403936	BetaCoV/Guangdong/20SF028/2020	L	GISAID
EPI_ISL_403937	BetaCoV/Guangdong/20SF040/2020	L	GISAID
EPI_ISL_406531	BetaCoV/Guangdong/20SF174/2020	L	GISAID
EPI_ISL_406538	BetaCoV/Guangdong/20SF201/2020	L	GISAID
EPI_ISL_406862	BetaCoV/Germany/BavPat1/2020	L	GISAID
EPI_ISL_406533	BetaCoV/Guangzhou/20SF206/2020	L	GISAID
EPI_ISL_407313	BetaCoV/Hangzhou/HZCDC0001/2020	L	GISAID
EPI_ISL_406970	BetaCoV/Hangzhou/HZ-1/2020	L	GISAID
EPI_ISL_407084	BetaCoV/Japan/AI/I-004/2020	L	GISAID
EPI_ISL_408669	BetaCoV/Japan/KY-V-029/2020	L	GISAID
EPI_ISL_408488	BetaCoV/Jiangsu/IVDC-JS-001/2020	L	GISAID
EPI_ISL_408486	BetaCoV/Jiangxi/IVDC-JX-002/2020	L	GISAID
EPI_ISL_410301	BetaCoV/Nepal/61/2020	L	GISAID
EPI_ISL_408482	BetaCoV/Shandong/IVDC-SD-001/2020	L	GISAID
EPI_ISL_406973	BetaCoV/Singapore/1/2020	L	GISAID
EPI_ISL_407987	BetaCoV/Singapore/2/2020	L	GISAID
EPI_ISL_407988	BetaCoV/Singapore/3/2020	L	GISAID
EPI_ISL_411929	BetaCoV/South Korea/SNU01/2020	L	GISAID

EPI_ISL_408976	BetaCoV/Sydney/2/2020	L	GISAID
EPI_ISL_408977	BetaCoV/Sydney/3/2020	L	GISAID
EPI_ISL_406594	BetaCoV/Shenzhen/SZTH-003/2020	L	GISAID
EPI_ISL_406595	BetaCoV/Shenzhen/SZTH-004/2020	L	GISAID
EPI_ISL_403962	BetaCoV/Nonthaburi/61/2020	L	GISAID
EPI_ISL_403963	BetaCoV/Nonthaburi/74/2020	L	GISAID
EPI_ISL_406031	BetaCoV/Taiwan/2/2020	L	GISAID
EPI_ISL_410218	BetaCov/Taiwan/NTU02/2020	L	GISAID
EPI_ISL_406036	BetaCoV/USA/CA2/2020	L	GISAID
EPI_ISL_410044	BetaCoV/USA/CA6/2020	L	GISAID
EPI_ISL_408010	BetaCoV/USA/CA5/2020	L	GISAID
EPI_ISL_409067	BetaCoV/USA/MA1/2020	L	GISAID
EPI_ISL_408009	BetaCoV/USA/CA4/2020	L	GISAID
EPI_ISL_408008	BetaCoV/USA/CA3/2020	L	GISAID
EPI_ISL_408670	BetaCoV/USA/WI1/2020	L	GISAID
EPI_ISL_402123	BetaCoV/Wuhan/IPBCAMS-WH-01/2019	L	GISAID
EPI_ISL_406798	BetaCov/Wuhan/WH01/2019	L	GISAID
EPI_ISL_402127	BetaCoV/Wuhan/WIV02/2019	L	GISAID
EPI_ISL_402124	BetaCoV/Wuhan/WIV04/2019	L	GISAID
EPI_ISL_402128	BetaCoV/Wuhan/WIV05/2019	L	GISAID
EPI_ISL_402129	BetaCoV/Wuhan/WIV06/2019	L	GISAID
EPI_ISL_402130	BetaCoV/Wuhan/WIV07/2019	L	GISAID
EPI_ISL_403931	BetaCoV/Wuhan/IPBCAMS-WH-02/2019	L	GISAID
EPI_ISL_403930	BetaCoV/Wuhan/IPBCAMS-WH-03/2019	L	GISAID
EPI_ISL_403929	BetaCoV/Wuhan/IPBCAMS-WH-04/2019	L	GISAID
EPI_ISL_402132	BetaCoV/Wuhan/HBCDC-HB-01/2019	L	GISAID
EPI_ISL_402119	BetaCoV/Wuhan/IVDC-HB-01/2019	L	GISAID
EPI_ISL_402121	BetaCoV/Wuhan/IVDC-HB-05/2019	L	GISAID
EPI_ISL_402125	BetaCoV/Wuhan-Hu-1/2019	L	GISAID
EPI_ISL_406800	BetaCov/Wuhan/WH02/2019	L	GISAID
EPI_ISL_403928	BetaCoV/Wuhan/IPBCAMS-WH-05/2020	L	GISAID

EPI_ISL_402120	BetaCoV/Wuhan/IVDC-HB-04/2020	L	GISAID
EPI_ISL_408514	BetaCoV/Wuhan/IVDC-HB-envF13-20/2020	L	GISAID
EPI_ISL_408515	BetaCoV/Wuhan/IVDC-HB-envF13-21/2020	L	GISAID
EPI_ISL_406716	BetaCoV/China/WHU01/2020	L	GISAID
EPI_ISL_406717	BetaCoV/China/WHU02/2020	L	GISAID
EPI_ISL_404227	BetaCoV/Zhejiang/WZ-01/2020	L	GISAID
EPI_ISL_404228	BetaCoV/Zhejiang/WZ-02/2020	L	GISAID
NMDC60013002-09	BetaCoV/Wuhan/WH19004/2020	L	NMDC
NMDC60013002-07	BetaCoV/Wuhan/YS8011/2020	L	NMDC
NMDC60013002-06	BetaCoV/Wuhan/WH19008/2019	L	NMDC
NMDC60013002-08	BetaCoV/Wuhan/WH19001/2019	L	NMDC
NMDC60013002-10	BetaCoV/Wuhan/WH19005/2019	L	NMDC
EPI_ISL_407893	BetaCoV/Australia/NSW01/2020	S	GISAID
EPI_ISL_407894	BetaCoV/Australia/QLD01/2020	S	GISAID
EPI_ISL_407896	BetaCoV/Australia/QLD02/2020	S	GISAID
EPI_ISL_407976	BetaCoV/Belgium/GHB-03021/2020	S	GISAID
EPI_ISL_408478	BetaCoV/Chongqing/YC01/2020	S	GISAID
EPI_ISL_407071	BetaCoV/England/01/2020	S	GISAID
EPI_ISL_407073	BetaCoV/England/02/2020	S	GISAID
EPI_ISL_403932	BetaCoV/Guangdong/20SF012/2020	S	GISAID
EPI_ISL_403935	BetaCoV/Guangdong/20SF025/2020	S	GISAID
EPI_ISL_403933	BetaCoV/Guangdong/20SF013/2020	S	GISAID
EPI_ISL_408665	BetaCoV/Japan/TY-WK-012/2020	S	GISAID
EPI_ISL_408667	BetaCoV/Japan/TY-WK-521/2020	S	GISAID
EPI_ISL_408666	BetaCoV/Japan/TY-WK-501/2020	S	GISAID
EPI_ISL_407193	BetaCoV/South Korea/KCDC03/2020	S	GISAID
EPI_ISL_408484	BetaCoV/Sichuan/IVDC-SC-001/2020	S	GISAID
EPI_ISL_406030	BetaCoV/Shenzhen/HKU-SZ-002/2020	S	GISAID
EPI_ISL_405839	BetaCoV/Shenzhen/HKU-SZ-005/2020	S	GISAID
EPI_ISL_406593	BetaCoV/Shenzhen/SZTH-002/2020	S	GISAID
EPI_ISL_408489	BetaCoV/Taiwan/NTU01/2020	S	GISAID

EPI_ISL_404895	BetaCoV/USA/WA1/2020	S	GISAID
EPI_ISL_406223	BetaCoV/USA/AZ1/2020	S	GISAID
EPI_ISL_406034	BetaCoV/USA/CA1/2020	S	GISAID
EPI_ISL_407214	BetaCoV/USA/WA1-A12/2020	S	GISAID
EPI_ISL_407215	BetaCoV/USA/WA1-F6/2020	S	GISAID
EPI_ISL_410045	BetaCoV/USA/IL2/2020	S	GISAID
EPI_ISL_408668	BetaCoV/Vietnam/VR03-38142/2020	S	GISAID
EPI_ISL_406801	BetaCov/Wuhan/WH04/2020	S	GISAID
MT049951	SARS-CoV-2/Yunnan-01/human/2020/CHN	S	GenBank
EPI_ISL_408480	BetaCoV/Yunnan/IVDC-YN-003/2020	S	GISAID
EPI_ISL_404253	BetaCoV/USA/IL1/2020	L+S	GISAID
EPI_ISL_406592	BetaCoV/Shenzhen/SZTH-001/2020	Unknown	GISAID

*GISAID (Global Initiative on Sharing All Influenza Data; https:/www.gisaid.org/) has published a compilation of 103 full genome sequences with recognition, GenBank (https:/www.ncbi.nlm.nih.gov /genbank), and NMDC (National Microbiology Data Center; http:/nmdc.cn/#/nCoV).
